# Machine learning based hepatic safety score predicts decompensation in hepatocellular carcinoma systemic therapy

**DOI:** 10.1038/s41746-026-02802-3

**Published:** 2026-05-22

**Authors:** Ji Won Han, Jaejun Lee, Keungmo Yang, Kwon Yong Tak, Hyun Yang, Si Hyun Bae, Hee Sun Cho, Heechul Nam, Chang Wook Kim, Hae Lim Lee, Hee Yeon Kim, Sung Won Lee, Ahlim Lee, Do Seon Song, Seok Hwan Kim, Myeong Jun Song, Soon Woo Nam, Soon Kyu Lee, Jung Hyun Kwon, Pil Soo Sung, Jeong Won Jang

**Affiliations:** 1 The Catholic University Liver Research Center, Department of Biomedicine & Health Sciences, College of Medicine, The Catholic University of Korea, Seoul, Republic of Korea; 2 Division of Hepatology, Department of Internal Medicine, Seoul St. Mary’s Hospital, College of Medicine, The Catholic University of Korea, Seoul, Republic of Korea; 3 Division of Hepatology, Department of Internal Medicine, Eunpyeong St. Mary’s Hospital, College of Medicine, The Catholic University of Korea, Seoul, Republic of Korea; 4 Division of Hepatology, Department of Internal Medicine, Uijeongbu St. Mary’s Hospital, College of Medicine, The Catholic University of Korea, Seoul, Republic of Korea; 5 Division of Hepatology, Department of Internal Medicine, Bucheon St. Mary’s Hospital, College of Medicine, The Catholic University of Korea, Seoul, Republic of Korea; 6 Division of Hepatology, Department of Internal Medicine, St. Vincent Hospital, College of Medicine, The Catholic University of Korea, Seoul, Republic of Korea; 7 Division of Hepatology, Department of Internal Medicine, Daejeon St. Mary’s Hospital, College of Medicine, The Catholic University of Korea, Seoul, Republic of Korea; 8 Division of Hepatology, Department of Internal Medicine, Incheon St. Mary’s Hospital, College of Medicine, The Catholic University of Korea, Seoul, Republic of Korea

**Keywords:** Cancer, Diseases, Gastroenterology, Oncology

## Abstract

Hepatocellular carcinoma (HCC) frequently coexists with portal hypertension, significantly increasing the risk of hepatic decompensation (HD) and variceal bleeding during systemic therapy. We developed a machine learning based hepatic safety score (MHSS) using data from 2026 patients with unresectable HCC to predict clinically significant portal hypertension (CSPH) and prognosis. A random forest model was trained in a derivation cohort (n = 1262) and validated in an independent cohort (n = 764). The MHSS demonstrated robust performance (AUROC 0.840) in CSPH and predicting HD. Stratification revealed that high MHSS patients faced significantly elevated risks of HD (HR 3.25), variceal bleeding (VB, HR 4.90), and mortality (HR 2.21). Crucially, while atezolizumab-bevacizumab offered a survival advantage in low MHSS patients, it was associated with high bleeding risk and no survival benefit in the high MHSS group compared to other regimens. A simulation of MHSS guided treatment selection demonstrated a 24% reduction in HD, a 40% reduction in VB, and a 26% reduction in mortality. In conclusion, the MHSS effectively predicts CSPH, decompensation, and survival in patients with HCC prior to systemic therapy. By enabling individualized risk stratification, the MHSS may guide personalized treatment selection between bevacizumab-containing and alternative regimens, ultimately improving patient outcomes. Clinical trial number: not applicable.

## Introduction

The emergence of combination immunotherapy with atezolizumab plus bevacizumab (Atezo-bev) has significantly improved survival in unresectable hepatocellular carcinoma (HCC), establishing a new first-line standard^1^.

Despite these advances, advanced HCC still has high mortality^1^, and further improvements hinge not only on tumor control but also on preserving liver function during therapy. A key challenge in treating HCC patients with systemic therapies is the underlying cirrhosis and risk of hepatic decompensation (HD). HD-defined by complications such as ascites, variceal bleeding (VB), or hepatic encephalopathy (HE)-has emerged as a critical prognostic factor in advanced HCC. A recent trial showed that onset of HD within 3 months of starting therapy was associated with a hazard ratio (HR) ~ 7.6 for death, exceeding the mortality risk of radiologic tumor progression (HR ~ 5.9) in Atezo-bev treated patients^2^. Similarly, a large real-world cohort of Atezo-bev treated patients also found that those who developed HD had a dramatically worse prognosis, with HD conferring an HR ~ 19 for mortality versus those without HD^3^. These data underscore that HD can outweigh tumor progression as a driver of outcomes in advanced HCC receiving systemic treatments including Atezo-bev. In addition to its direct impact on survival, HD frequently leads to treatment discontinuation, which in turn could limit the effectiveness of tumor control.

Portal hypertension is a central mechanism underlying HD in cirrhosis, and its presence portends the development of complications. Clinically significant portal hypertension (CSPH) is defined by a hepatic venous pressure gradient (HVPG) ≥ 10 mmHg, and this threshold strongly predicts the onset of VB, ascites, and other decompensating events^4^. However, direct HVPG measurement is an invasive catheter-based procedure requiring specialized expertise and is seldom feasible in HCC patients outside of research settings. Thus, while HVPG is the gold-standard gauge of portal pressure, its limitations have spurred interest in non-invasive surrogates to identify CSPH in patients at risk. Several alternative methods are used to infer portal hypertension and risk of VB in cirrhosis without routine HVPG. Esophagogastroduodenoscopy (EGD) remains the clinical standard for detecting esophageal varices^5^, which are a consequence of CSPH. Cross-sectional imaging such as contrast-enhanced computed tomography (CT) or magnetic resonance imaging (MRI) can also reveal stigmata of CSPH-including venous collaterals, splenomegaly, and varices-that also correlate with HVPG^6^. While liver function tests-based scores such as ALBI are not direct measures of portal pressure, they reflect the degree of hepatic impairment and have some association with HD risk^7^. Non-invasive liver stiffness measurement also might be helpful^8^, which might be limited by the tumor involvement in the liver parenchyma. Of note, while previous studies have developed non-invasive models for predicting CSPH in compensated cirrhosis^9,10^, there remains a lack of robust tools to quantitatively integrate all relevant factors and predict CSPH and impending HD in the context of systemic therapies in HCC patients.

The 2023 AASLD Practice Guidance recommends endoscopic evaluation for high-risk varices prior to Atezo-bev initiation, with consideration of durvalumab-tremelimumab (Durva-treme) for patients with high bleeding risk^11^. Non-invasive tools to identify patients at risk of portal hypertension-related complications could complement these recommendations by enabling targeted screening and personalized treatment selection.

Therefore, the aim of this study was to develop and validate a machine learning–based Hepatic Safety Score (MHSS) that integrates tumor burden and laboratory indicators to assess clinically significant portal hypertension (CSPH). Specifically, we sought to non-invasively identify baseline CSPH—defined by the presence of varices, radiologic ascites, or imaging findings—and to determine whether this classification could predict subsequent HD and overall prognosis in patients with advanced HCC receiving systemic therapy, including Atezo-bev.

## Results

### Predictive performance of the MHSS

Baseline characteristics of the derivation (n = 1262) and validation cohorts (n = 764) are demonstrated in Table S1. The MHSS was developed using an RF algorithm to predict CSPH, since the RF model consistently exhibited superior diagnostic performance for CSPH compared to other ML algorithms tested, achieving the highest AUROC of 0.840 and 0.802 in the derivation and validation cohorts, respectively (Table S2), and other algorithms including XGBoost and gradient boosting also demonstrated modest predictive capacities; however, none exceeded the performance of the RF model.

In further analyses comparing MHSS with established non-invasive scores including Fibrosis-4 index, AST to platelet ratio index, Albumin-Bilirubin (ALBI) grade, modified ALBI grade, Child-Pugh score, and Model for End-stage Liver Disease (Table 1), MHSS demonstrated significantly higher predictive accuracy for HD at multiple time points (3, 6, and 12 months), consistently attaining AUROC values greater than 0.8 (3-month AUROC: 0.813; 6-month AUROC: 0.804; 12-month AUROC: 0.812). Although ALBI score approached MHSS performance at the 3- and 6-months incident HD, MHSS retained statistically significant superiority at 12-month (AUROC: 0.765, p = 0.028). Center-stratified analysis demonstrated consistent MHSS performance across all 8 hospitals (Fig. S1), supporting multi-center robustness. Calibration analysis demonstrated good agreement between predicted and observed probabilities, with Brier scores of 0.261 for CSPH, 0.259 for 3-month, 0.211 for 6-month, and 0.169 for 12-month HD (Fig. S2). These findings highlight the robust predictive advantage of MHSS for accurately predicting CSPH and future incident HD in advanced HCC.

**Table 1:** Comparison of prediction performances for hepatic decompensation

	3-month Decompensation (n = 623, 151 events)	6-month Decompensation (n = 547, 215 events)	12-month Decompensation (n = 424, 271 events)
MHSS	0.813 (Reference)	0.804 (Reference)	0.812 (Reference)
FIB-4	0.708 (<0.001)	0.712 (<0.001)	0.733 (<0.001)
APRI	0.727 (<0.001)	0.727 (<0.001)	0.750 (0.005)
ALBI	0.800 (0.459)	0.784 (0.333)	0.765 (0.028)
mALBI	0.761 (0.006)	0.746 (0.003)	0.736 (0.001)
MELD	0.567 (<0.001)	0.554 (<0.001)	0.567 (<0.001)
Child-Pugh	0.754 (0.003)	0.741 (0.001)	0.725 (<0.001)

Values are presented as AUROC (p value compared to MHSS). AUROC: Area Under the Receiver Operating Characteristic Curve. P values were calculated using the DeLong test for comparison of AUROC between each model and the MHSS.

*MHSS* machine-learning-based Hepatic Safety Score, *FIB-4* fibrosis-4 index, *APRI* AST to Platelet Ratio Index, *ALBI* Albumin-Bilirubin grade, *MELD* model for End-stage Liver Disease.

### Clinical characteristics of patients stratified by MHSS

Feature importance analysis using Shapley additive explanation (SHAP) values (Fig. S3) demonstrated that the predictive accuracy of MHSS was based on a combination of conventional liver function parameters (total bilirubin, INR, AST, albumin, platelet count) and key tumor-related variables, notably largest tumor size, alpha-fetoprotein (AFP), and protein induced by vitamin K absence or antagonist-II (PIVKA-II). SHAP dependence plots showed expected directional relationships with CSPH risk prediction (Fig. S4).

We next classified patients among the validation cohort into high (37.8%) and low (62.2%) MHSS groups according to an optimized cutoff, and compared their baseline characteristics (Table 2). As a result, patients in the high MHSS group displayed significantly poorer clinical features, characterized by larger intrahepatic tumor size (median 9.0 cm vs. 5.0 cm; p < 0.001), more frequent multiple tumors (88.6% vs. 71.8%; p < 0.001) and PVT (66.4% vs. 31.8%; p < 0.001), and elevated tumor markers (AFP median 1,410.0 vs. 97.7 ng/mL, PIVKA-II median 5,523.2 vs. 806.0 mAU/mL; p < 0.001). Additionally, significantly poorer liver function was evident in the high MHSS group, indicated by higher levels of AST and total bilirubin, decreased albumin levels, and lower platelet counts. These findings suggest that MHSS effectively integrates tumor factors and liver dysfunction, both of which are critical determinants of decompensation and prognosis in patients with advanced HCC.

**Table 2:** Baseline characteristics according to MHSS group

	Low MHSS (n = 475)	High MHSS (n = 289)	p value
Demographics			
Male sex	399/475 (84.0%)	249/289 (86.2%)	0.411
Age, years	64.3 ± 12.2	62.3 ± 10.8	0.007
Etiology			
HBV	303/475 (63.8%)	191/289 (66.1%)	0.521
HCV	31/475 (6.5%)	24/289 (8.3%)	0.364
Alcohol	114/475 (24.0%)	95/289 (32.9%)	0.008
Others	77/475 (16.2%)	26/289 (9.0%)	0.004
Largest intrahepatic size, cm	5.0 (1.8–9.5)	9.0 (4.3–13.0)	<0.001
Multiple tumors	341/475 (71.8%)	256/289 (88.6%)	<0.001
PVT	151/475 (31.8%)	192/289 (66.4%)	<0.001
EHS	338/475 (71.2%)	152/289 (52.8%)	<0.001
AST, U/L	40 (28–68)	81 (52–126)	<0.001
ALT, U/L	25 (17–41)	36 (23–57)	<0.001
Total bilirubin, mg/dL	0.6 (0.5–0.8)	1.4 (1.0–1.8)	<0.001
Albumin, g/dL	3.9 ± 0.5	3.5 ± 0.5	<0.001
Platelet, ×10^3^/μL	169 (124–229)	127 (85–204)	<0.001
INR	1.1 (1.0–1.1)	1.2 (1.1–1.3)	<0.001
AFP, ng/mL	97.7 (6.7–2,634.0)	1,410.0 (109.0–13,750.0)	<0.001
PIVKA-II, mAU/mL	806.0 (70.5–8,322.6)	5,523.2 (566.0–30,980.0)	<0.001

Continuous variables are presented as mean ± SD or median (interquartile range) according to data distribution. P values < 0.001 are shown as “<0.001”, and all other p-values are shown to three decimal places.

*MHSS* machine-learning-based hepatic safety score, *HBV* Hepatitis B virus, *HCV* Hepatitis C virus, *PVT* portal vein thrombosis, *EHS* extrahepatic spread, *AST* aspartate aminotransferase, *ALT* alanine aminotransferase, *INR* international normalized ratio, *AFP* alpha-fetoprotein, *PIVKA-II* protein induced by vitamin K absence or antagonist-II.

### Prognostic significance of MHSS for HD, VB, and OS

We next performed Kaplan-Meier survival analyses (Fig. S5), which revealed a strong correlation between early liver-related events (HD and VB within 3 months) and reduced OS. Specifically, patients who experienced HD within 3 months had a markedly decreased OS, with a HR of 4.85 (95% CI 3.28–7.19; p < 0.001). Similarly, patients with early VB also showed a significantly increased risk of mortality (HR = 4.35, 95% CI 1.89–10.03; p < 0.001). These findings emphasize the prognostic impact of HD and the need for early identification of at-risk individuals.

**Fig. 1:**
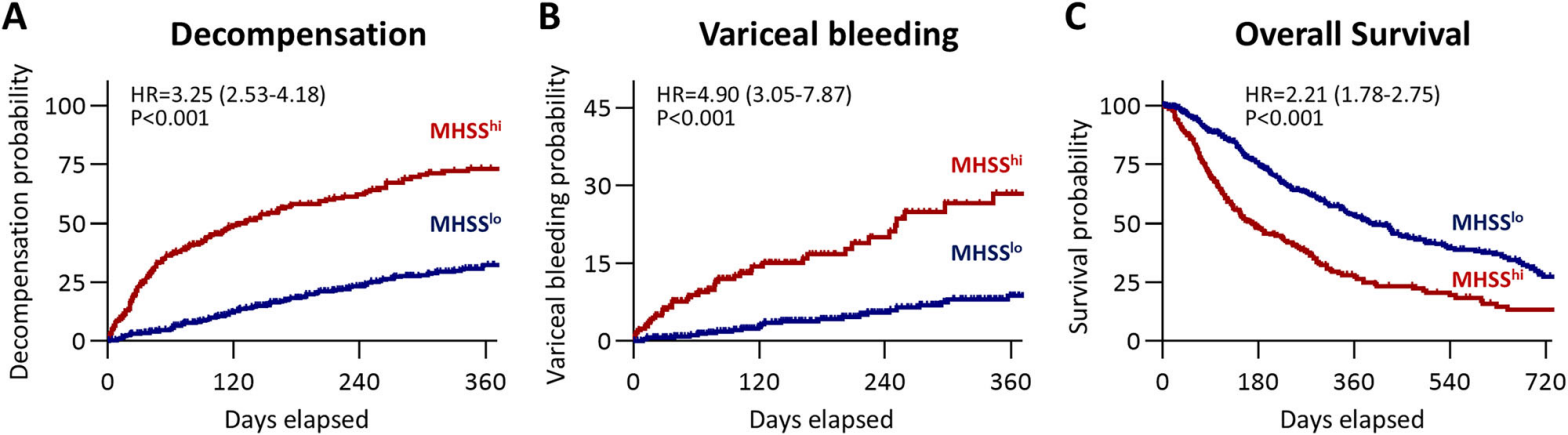
Kaplan-Meier curves comparing clinical outcomes between high MHSS (MHSShi, n = 289) and low MHSS (MHSSlo, n = 475) groups. **A** Cumulative incidence of hepatic decompensation. **B** Cumulative incidence of variceal bleeding. **C** Overall survival probability. MHSS machine-learning-based Hepatic Safety Score.

Further Kaplan-Meier analyses stratified by MHSS classification (Fig. 1) demonstrated that patients in the high MHSS group had consistently worse clinical outcomes than those in the low MHSS group. The high MHSS group exhibited a significantly increased cumulative risk of HD (HR = 3.25, 95% CI 2.53–4.18; p < 0.001), VB (HR = 4.90, 95% CI 3.05–7.87; p < 0.001), and reduced OS (HR = 2.21, 95% CI 1.78–2.75; p < 0.001). The cumulative incidence of HD in the high MHSS group was 42.5% at 3 months, 58.0% at 6 months, and 73.2% at 12 months, compared to 8.6%, 18.2%, and 32.4% in the low MHSS group. For VB, the cumulative incidence was also significantly higher in the high MHSS group: 11.95%, 16.78%, and 28.46% at 3, 6, and 12 months, respectively, versus 1.86%, 4.22%, and 8.76% in the low MHSS group. Sensitivity analysis excluding patients who received prophylactic endoscopic variceal ligation (EVL) demonstrated that the MHSS maintained robust predictive performance for VB (n = 739, HR = 3.64, 95% CI 2.26–5.84, p < 0.001). Accordingly, OS at 3, 6, and 12 months was markedly lower in the high MHSS group (69.85%, 48.27%, and 26.50%) than in the low MHSS group (88.99%, 75.53%, and 52.83%). Subgroup analyses confirmed consistent MHSS performance for incident HD across line of therapy (1st-line: HR = 3.28; later-line: HR = 3.50), treatment history (naïve: HR = 3.14; experienced: HR = 3.24), etiology (viral: HR = 2.90; non-viral: HR = 4.34), and baseline liver function (Child-Pugh A: HR = 2.95; Child-Pugh B: HR = 1.99), all with p < 0.05 (Fig. S6). These results validate MHSS as a robust prognostic tool for identifying patients at increased risk for both HD and mortality.

To evaluate whether MHSS provides prognostic information independent of tumor burden, propensity score matching (PSM) was performed based on tumor size, portal vein thrombosis, extrahepatic spread, multiple tumors, and AFP (Table S3). After matching (n = 233 pairs), the MHSS maintained significant predictive value for HD (HR = 1.77, 95% CI 1.34–2.34, p < 0.001) and VB (HR = 1.91, 95% CI 1.10–3.30, p = 0.021), confirming that MHSS also captures hepatic reserve risk beyond tumor burden.

### MHSS-based treatment stratification and clinical outcomes

We next analyzed clinical outcomes under different systemic therapies-Atezo-bev, nivolumab, and lenvatinib-to identify potential treatment-specific benefits (Fig. S7). Although HD risks were similar across treatment groups, Atezo-bev exhibited higher VB incidence (p = 0.002) but notably better OS outcomes (p < 0.001) compared to the other two regimens.

**Fig. 2:**
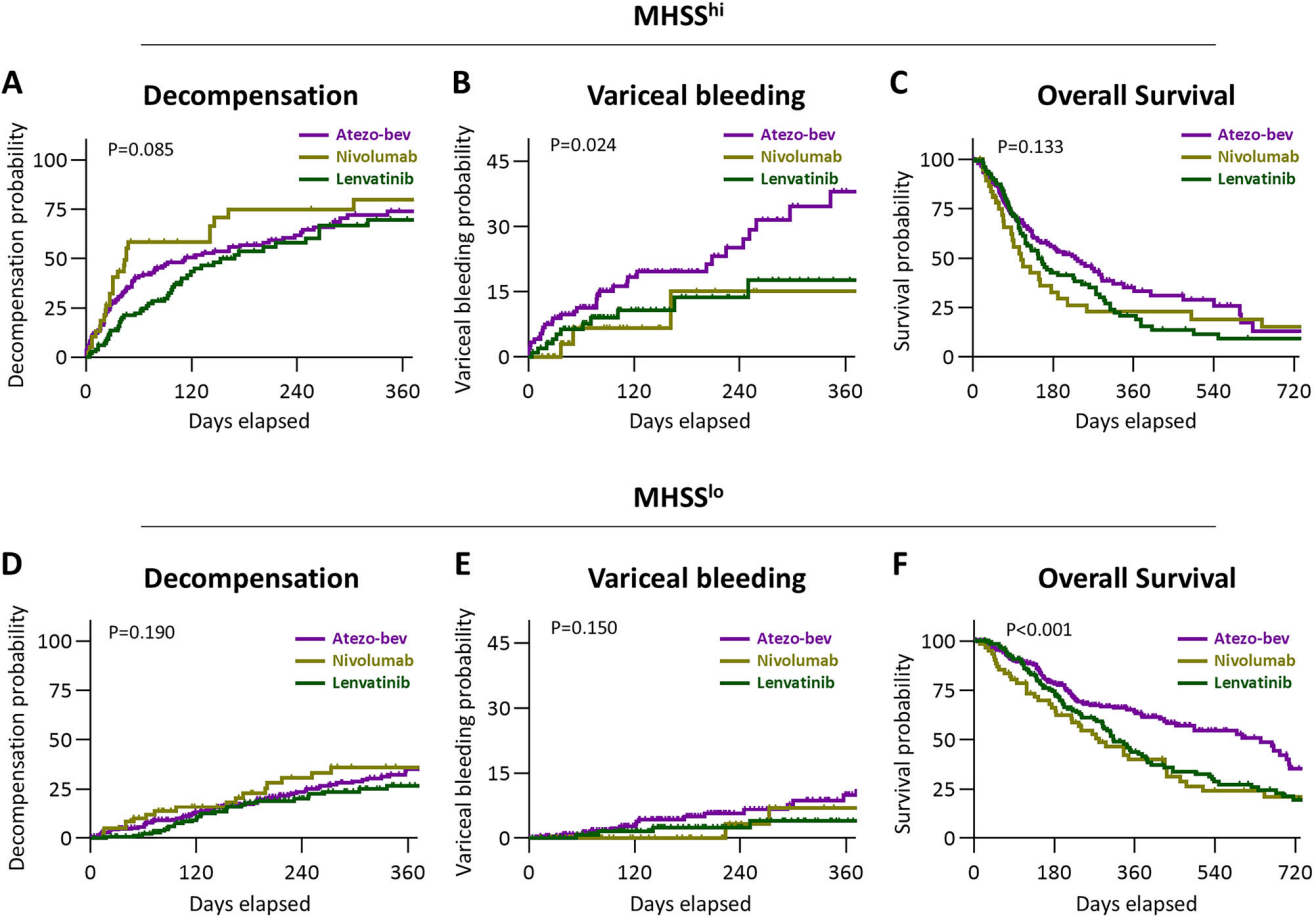
Clinical outcomes among treatment regimens stratified by MHSS classification. Comparison of treatment regimens in the high MHSS (MHSShi) group (Atezo-bev, n = 150, Nivolumab, n = 38, Lenvatinib, n = 101) showing Kaplan-Meier curves for: **A** hepatic decompensation probability, **B** variceal bleeding probability, and **C** overall survival. **D**–**F** Comparison in the low MHSS (MHSSlo) group (Atezo-bev, n = 262, Nivolumab, n = 62, Lenvatinib, n = 151): **D** hepatic decompensation probability, **E** variceal bleeding probability, and **F** overall survival. MHSS machine-learning-based Hepatic Safety Score, Atezo-bev Atezolizumab-bevacizumab.

Further stratification by MHSS classification revealed substantial differences in clinical outcomes between systemic therapies (Fig. 2). Clinical characteristics of each treatment group among high and low MHSS groups are presented in Table S4, demonstrating that there were no differences in clinical characteristics except having larger tumor size and more frequent PVT in Atezo-bev treated patients among low MHSS group. In the high MHSS group, the cumulative risk of HD (Fig. 2A) was not different between Atezo-bev and other regimens (p = 0.085). However, the cumulative risk of VB (Fig. 2B) was significantly higher in patients receiving Atezo-bev than those treated with nivolumab or lenvatinib (p = 0.024). Importantly, OS (Fig. 2C) was not significantly different among the three treatment groups in the high MHSS group (p = 0.133).

In the low MHSS group, the cumulative risk of HD (Fig. 2D) and VB (Fig. 2E) was comparable across treatment arms (p = 0.190 and p = 0.150, respectively). However, higher OS (Fig. 2F) was observed in patients treated with Atezo-bev compared to those receiving nivolumab or lenvatinib (p < 0.001), despite this group having larger tumor size and more frequent PVT (Table S4). Multivariate Cox regression analysis adjusting for tumor size, PVT, extrahepatic spread (EHS), multiple tumors, AFP, and treatment duration confirmed that the survival advantage of Atezo-bev in low MHSS patients was independent of these confounders (Table S5). These results suggest the discriminatory power of MHSS in predicting treatment-specific outcomes and highlight its utility in identifying patients most likely to benefit from Atezo-bev, while underscoring the increased VB risk associated with its use in high-risk patients.

Table 3 provides detailed HRs and statistical significance levels for HD, VB, and OS within each treatment subgroup. In the Atezo-bev cohort, high MHSS patients had significantly increased risks of HD (HR = 3.74, 95% CI 2.79–5.02; p < 0.001), VB (HR = 4.10, 95% CI 2.44–6.90; p < 0.001), and reduced OS (HR = 2.39, 95% CI 1.77–3.24; p < 0.001). In the lenvatinib group, high MHSS status was also associated with significantly worse outcomes, with increased risk of HD (HR = 3.73, 95% CI 2.45–5.67; p < 0.001), VB (HR = 5.08, 95% CI 1.91–13.53; p = 0.001), and shorter OS (HR = 1.89, 95% CI 1.38–2.59; p < 0.001). For nivolumab-treated patients, high MHSS similarly predicted increased risk of HD (HR = 2.85, 95% CI 1.60–5.06; p < 0.001), although associations with VB and OS were not statistically significant. These results underscore the prognostic value of MHSS across treatment modalities and its potential to inform therapy selection.

**Table 3:** Univariate cox regression analysis of hepatic decompensation, variceal bleeding, and overall survival according to MHSS status in different treatment subgroups

Treatment	Outcome	HR (95% CI)	p value
Lenvatinib	Hepatic Decompensation	3.73 (2.45–5.67)	<0.001
	Variceal Bleeding	5.08 (1.91–13.53)	0.001
	Overall Survival	1.89 (1.38–2.59)	<0.001
Nivolumab	Hepatic Decompensation	2.85 (1.60–5.06)	<0.001
	Variceal Bleeding	3.44 (0.82–14.41)	0.091
	Overall Survival	1.16 (0.72–1.88)	0.533
Atezo-bev	Hepatic Decompensation	3.74 (2.79–5.02)	<0.001
	Variceal Bleeding	4.10 (2.44–6.90)	<0.001
	Overall Survival	2.39 (1.77–3.24)	<0.001

Data are presented as hazard ratio (HR) with 95% confidence interval (CI). Comparison was performed between high and low MHSS groups.

### Simulation of MHSS-guided treatment selection

**Fig. 3:**
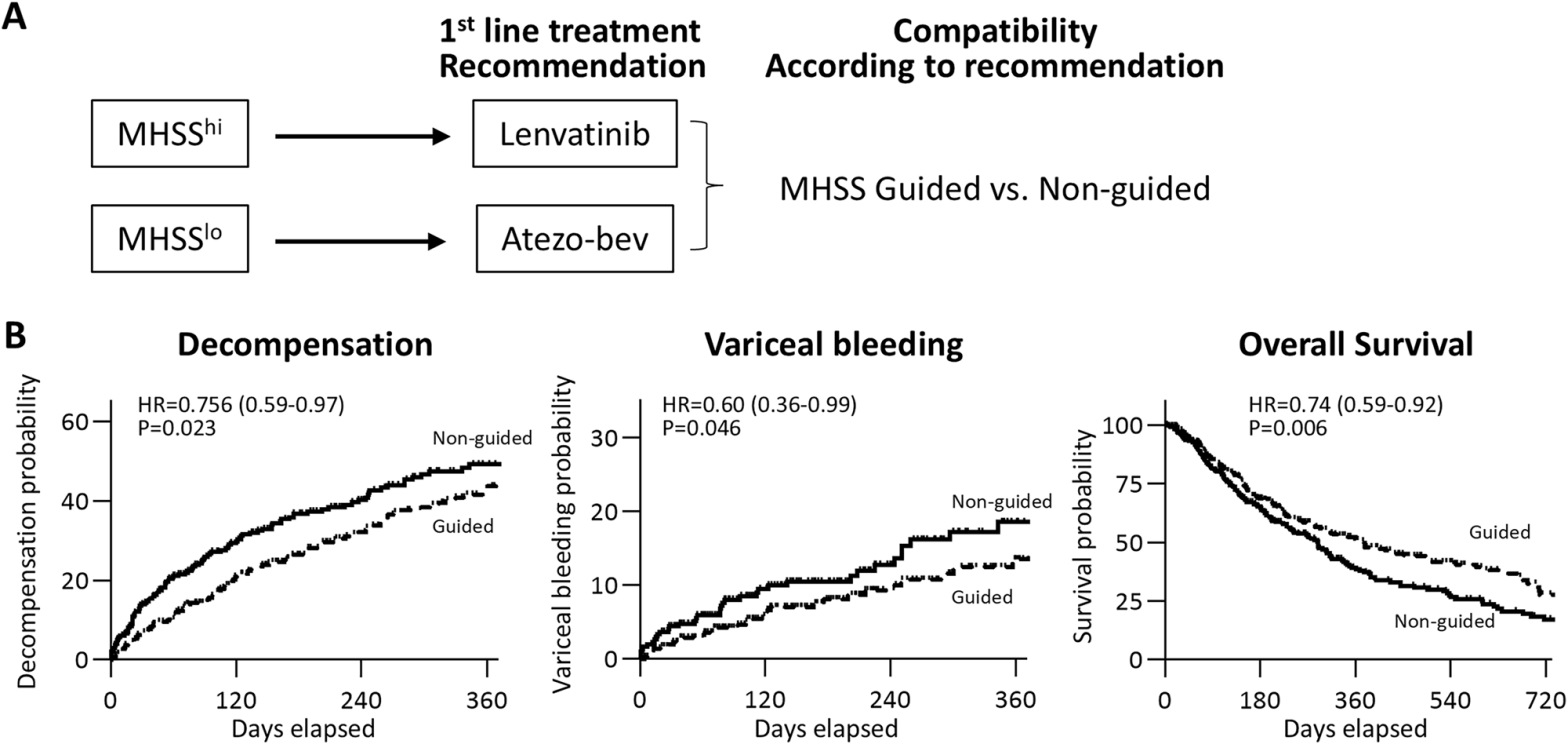
Simulation of the MHSS-guided treatment strategy. **A** Algorithm for treatment recommendation based on MHSS status (MHSS Guided, n = 363 vs. Non-guided, n = 301). **B** Kaplan-Meier curves comparing clinical outcomes between MHSS-guided and non-guided treatment approaches for hepatic decompensation. MHSS Machine-learning-based Hepatic Safety Score, Atezo-bev Atezolizumab-bevacizumab.

To test the potential clinical application of MHSS, we conducted a simulation assigning first-line treatments based on MHSS classification (Fig. 3A): Atezo-bev for low MHSS and lenvatinib for high MHSS patients. In this simulation, 764 patients from the validation cohort were reallocated into the MHSS-guided treatment strategy, and outcomes were compared with the actual treatments they received (non-guided group). As a result, patients managed according to the MHSS-guided approach showed significantly better clinical outcomes across all endpoints (Fig. 3B). The risk of HD was reduced by 24% (HR = 0.76, 95% CI 0.59–0.97; p = 0.023), and VB was reduced by 40% (HR = 0.60, 95% CI 0.36–0.99; p = 0.046) compared to the non-guided group. Most notably, OS was significantly better in the MHSS-guided group, with a 26% risk reduction (HR = 0.74, 95% CI 0.59–0.92; p = 0.006). These improvements in both HD and OS indicate that MHSS-guided treatment selection may offer a clinically meaningful framework for personalizing systemic therapy. In the PSM cohort (Table S6), the MHSS-guided strategy demonstrated significantly lower risk of HD, and improved OS compared to non-guided treatment. VB also showed a favorable trend (Fig. S8). The findings support the integration of MHSS into the decision-making for patients with advanced HCC to better balance efficacy and safety.

### Clinical implications of MHSS stratification in patients receiving Atezo-bev

**Fig. 4:**
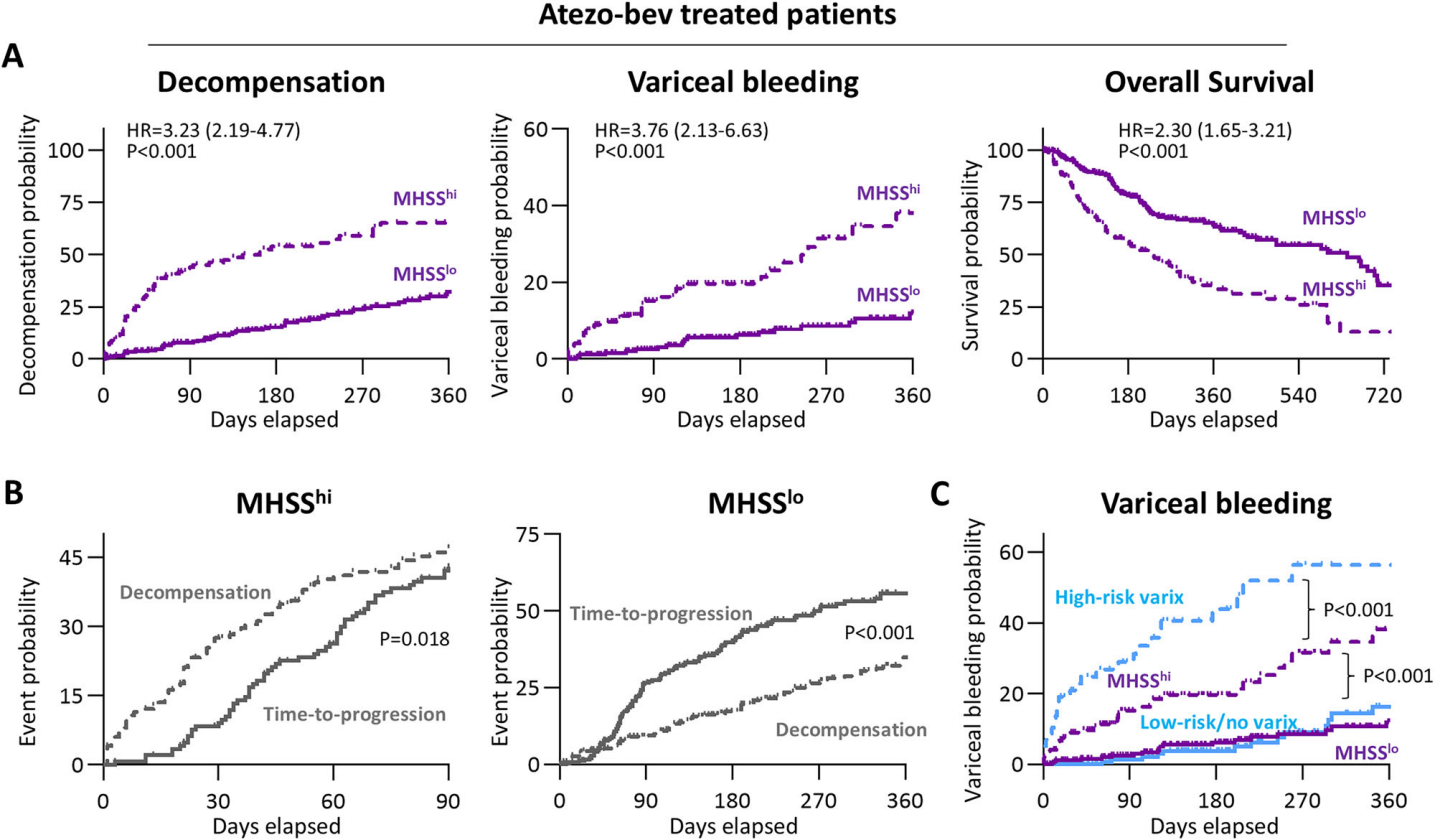
Clinical outcomes and competing risk analysis in atezolizumab-bevacizumab treated patients stratified by MHSS and varix status on endoscopy. **A** Kaplan-Meier survival curves comparing high MHSS (MHSShi, n = 150) versus low MHSS (MHSSlo, n = 262) groups in patients receiving Atezo-bev therapy, showing cumulative incidence of hepatic decompensation (left), variceal bleeding (middle), and overall survival probability (right). **B** Competing risk analysis between hepatic decompensation and time-to-progression in MHSShi (left) and MHSSlo (right) groups (n = 412). **C** Cumulative incidence of variceal bleeding stratified by baseline varix status on endoscopy (low-risk/no varix, n = 184, high-risk varix, n = 58) and MHSS classification (MHSShi, n = 150, MHSSlo, n = 262). MHSS machine-learning-based Hepatic Safety Score, Atezo-bev atezolizumab-bevacizumab.

Of note, in patients treated with Atezo-bev, MHSS classification clearly stratified clinical outcomes, clearly distinguishing risks for HD, VB, and OS in the Kaplan-Meier analyses (Fig. 4A). Furthermore, competing risk analyses further clarified distinct event sequences between groups (Fig. 4B); notably, in the high MHSS group, HD predominantly preceded radiologic tumor progression, strongly suggesting that HD in these patients occurs independently of-and is not merely a consequence of-tumor progression (p < 0.001). Conversely, patients in the low MHSS group more frequently exhibited tumor progression prior to HD (p = 0.018). Additionally, MHSS and baseline varix status assessed by EGD provided complementary risk stratification for VB (Fig. 4C). Patients with high-risk varices on EGD showed a significantly higher VB risk than the high MHSS group (p < 0.001), whereas patients in the low MHSS group had VB risks comparable to those with low-risk or no varices identified by EGD. These findings suggest that routine EGD could be safely spared in the low MHSS group, given their low VB risk. Conversely, patients in the high MHSS group should undergo mandatory EGD with consideration of prophylactic interventions to mitigate the elevated VB risk.

## Discussion

In this study, we developed and validated the MHSS, a novel machine learning-based model that predicts CSPH and hepatic decompensation in HCC patients undergoing systemic therapy. To our knowledge, this is the first ML-based tool specifically designed to prognosticate liver-related outcomes in the era of combination immunotherapy. The MHSS demonstrated superior predictive performance compared to conventional scoring systems, and consistently stratified patients into distinct risk groups for HD, VB, and OS across different treatment regimens. Unlike traditional scores that focus solely on liver function parameters, the MHSS uniquely integrates both hepatic reserve and tumor-related features, providing a comprehensive and personalized risk assessment. These findings address an important unmet clinical need, as portal hypertension-related complications remain a significant challenge during systemic therapy, particularly with bevacizumab-containing regimens.

A key strength of the MHSS is that it integrates both liver function parameters and tumor-related features to predict CSPH and HD risk. Traditional non-invasive scores like ALBI or Child-Pugh focus solely on liver dysfunction (albumin, bilirubin, INR, etc.) and neglect tumor characteristics^12^. However, tumor burden can significantly impact portal pressure and hepatic reserve in HCC. For instance, the presence of PVT has been shown to independently predict early HD in HCC patients on Atezo-bev therapy^2^. Similarly, real-world data indicate that HCC patients with PVT are at far higher risk of VB when treated with Atezo-bev (subdistribution HR ~ 3.5)^13^. Large tumors can also worsen liver function by replacing parenchyma or causing hepatic venous congestion, and these tumor effects are not captured by ALBI grade or Child-Pugh class^14^. Our ML model explicitly incorporated tumor-related variables alongside laboratory values; this ability to account for tumor-induced portal hypertension is a notable advance over prior scoring systems.

Our findings have significant clinical implications, particularly given the safety profile of current first-line therapies. Atezo-bev offers superior tumor control, but bevacizumab’s anti-VEGF effect predisposes patients to bleeding complications^15^. A recent multicenter study reported that approximately 7% of advanced HCC patients experienced VB within 6 months of starting Atezo-bev, despite pre-treatment endoscopic screening^13^. Importantly, that study identified factors like PVT, low platelets, and untreated varices as strong predictors of bleeding on Atezo-bev. These mirror many components of our MHSS model. Patients with high MHSS likely overlap with known high-risk groups, emphasizing the need for proactive prophylactic management^16^. Conversely, patients with a low MHSS (indicating low portal pressure and robust liver function) might proceed with Atezo-bev with more confidence and minimal modification. In essence, accurate prediction of CSPH and HD/VB risk allows clinicians to optimize supportive care and surveillance-for example, tailoring the frequency of monitoring for ascites, or early diuretic therapy to preempt overt decompensation.

Furthermore, our analysis further clarifies the clinical relevance of MHSS in guiding variceal management strategies. Specifically, in the Atezo-bev subgroup, we demonstrated that MHSS independently stratifies the risk of HD and VB, as well as their temporal relationship with tumor progression. Importantly, the competing risk analysis revealed that HD events occurred predominantly prior to tumor progression in patients classified as high MHSS, suggesting that liver-related events were not simply a consequence of advancing tumor burden. Additionally, MHSS effectively identified a low MHSS subgroup with a sufficiently low VB risk comparable to patients without high-risk varices detected on EGD. These findings provide clinical justification for potentially omitting routine EGD in low-risk patients, while strongly recommending endoscopic surveillance and prophylactic intervention in patients with high MHSS scores.

Moreover, the MHSS may inform the selection of systemic therapy in the first-line setting, especially now that multiple regimens are available. The latest treatment guidelines, including the updated Barcelona Clinic Liver Cancer (BCLC) strategy and international recommendations, have added durvalumab plus tremelimumab (Durva-treme) as an alternative first-line option, which is associated with lower rates of serious bleeding and predominantly immune-mediated liver toxicity rather than portal hypertensive events^17,18^. In clinical decision-making, physicians now must choose between Atezo-bev versus Durva-treme (or other options like tyrosine kinase inhibitors, TKIs) for a given patient. Our results, which stratified outcomes by MHSS risk levels suggest that the MHSS could play a role in this choice. For instance, a patient with a high MHSS might derive less net benefit from Atezo-bev due to the elevated risk of HD or VB. For such a patient, opting for Durva-treme or TKIs could be safer while still providing anti-tumor efficacy^19^. Thus, our MHSS-based risk stratification aligns with a personalized treatment approach, wherein high-risk patients avoid therapies that might precipitate liver-related events, and lower-risk patients receive the most effective regimen unhindered. In support of this approach, the MHSS-guided treatment simulation was based on our observation that Atezo-bev was associated with higher VB risk compared to lenvatinib or nivolumab in high MHSS patients, aligning with guideline recommendations for bevacizumab-free regimens in patients with high bleeding risk^11^. Our analysis showed that high MHSS patients had significantly greater rates of VB under Atezo-bev, whereas those treated with lenvatinib or nivolumab had fewer events. Taken together, the MHSS could be used alongside BCLC guidelines to guide first-line therapy: e.g. Figure 3 can be interpreted as a decision aid, where above a certain MHSS threshold, Durva-treme might be favored over Atezo-bev to improve safety.

We acknowledge several limitations of our study. First, the retrospective design and heterogeneous treatment periods across participating centers may introduce unmeasured confounders such as variations in supportive care practices across centers, although our large cohort and validation strengthen generalizability. Second, we lacked a dedicated validation cohort of patients treated with the newer Durva-treme regimen. However, we attempted to address this gap by testing the MHSS retrospectively in a subset of patients who received immunotherapy only (nivolumab). In our exploratory analysis, the MHSS retained prognostic ability in a nivolumab-treated cohort, suggesting that the score is capturing patient-intrinsic risk of decompensation rather than therapy-specific risk. If prospectively validated, MHSS could provide a non-invasive risk stratification strategy prior to treatment selection, potentially guiding decisions regarding EGD screening and choice between Atezo-bev and Durva-treme. Third, our study included patients with varying prior treatment histories and lines of therapy, which may confound treatment comparisons, although this reflects real-world clinical practice. Fourth, prophylactic management of VB and decompensation care were not standardized across centers, potentially affecting outcome interpretations. Prospective validation of MHSS is warranted to ensure it reliably improves clinical decision-making and to establish its utility in guiding therapy selection. Our study population was limited to Korean patients from a single healthcare network. Although subgroup analyses demonstrated consistent MHSS performance across viral and non-viral etiologies, external validation in geographically and ethnically diverse populations is warranted. To facilitate this, we have made a web-based MHSS calculator freely available. Despite these limitations, we attempted a rigorous approach with competing-risk analyses and sensitivity checks to ensure the robustness of our conclusions. Future studies incorporating HVPG measurements or elastography-based parameters may further enhance the predictive accuracy of portal hypertension assessment in HCC patients.

In conclusion, we developed and validated a machine learning-based model that quantitatively predicts CSPH and the risk of HD and VB in patients with advanced HCC receiving systemic therapy. By integrating tumor burden, liver function, and radiologic indicators into a single prognostic score, the MHSS enables individualized risk stratification with direct clinical relevance. This tool can assist in selecting appropriate therapies, planning surveillance strategies, and implementing prophylactic interventions. Ultimately, the MHSS may help optimize treatment and supportive care to improve survival while minimizing liver-related complications in the evolving landscape of HCC management.

## Methods

### Study patients

This study was approved by the Institutional Review Board of the Catholic University of Korea (IRB No. XC22RADI0014) and performed in accordance with the ethical principles of the Declaration of Helsinki. The requirement for informed consent was waived by the Institutional Review Board due to the retrospective nature of the study and the use of de-identified data. A total of 2026 patients were identified through medical record reviews conducted at eight affiliated hospitals between January 2010 and December 2024. Study flow is presented in Fig. S9. Eligible patients included those with HCC presenting with portal vein thrombosis (PVT), EHS, or an Eastern Cooperative Oncology Group (ECOG) performance status score of 1–2, consistent with BCLC-C^20^. This population was selected as they represent the primary candidates for systemic therapy and are at highest risk for portal hypertension-related complications due to advanced tumor burden and underlying cirrhosis. Patients were excluded if they had incomplete clinical data, concurrent non-HCC malignancies, Child-Pugh class C liver function, or ECOG performance status greater than 2. The diagnosis of HCC was established using imaging modalities such as CT or MRI, following the guideline for HCC management.21 Standard dosing protocols were followed: atezolizumab 1200 mg + bevacizumab 15 mg/kg Q3W, sorafenib 400 mg twice daily, lenvatinib based on body weight, nivolumab 3 mg/kg Q2W. Treatment modifications for toxicity followed the guideline^21^. Radiologic follow-up was performed using contrast-enhanced CT or MRI at baseline and at regular intervals (every 6–12 weeks), following institutional protocols aligned with the guideline^21^.

All patients underwent baseline contrast-enhanced CT or MRI, and 241 of 2,026 patients (11.9%) underwent baseline EGD. All eight participating centers are part of the Catholic Medical Center network and share a unified PACS and standardized imaging protocols, ensuring consistency in the assessment of portal hypertension-related findings across institutions. Esophagogastric varices were graded using a standardized classification system, and red color signs were systematically documented. Among patients who underwent pre-treatment EGD, those with high-risk varices received prophylactic EVL or non-selective beta-blockers according to institutional protocols.

### Definitions

CSPH was defined based on the identification of gastroesophageal varices on EGD or CT/MRI, the presence of spontaneous portosystemic shunts (SPSS), or the detection of ascites on imaging, as described in the previous report^6^. HD was defined as the development of new or worsening ascites, VB, or overt hepatic encephalopathy (West Haven grade ≥II).

Time-to-progression was defined as time to radiological progression per RECIST 1.1, and time-to-decompensation as time to the new onset or clinical worsening of ascites, VB, HE, or with death treated as a competing event. While tumor factors contribute to decompensation risk, the earlier occurrence of decompensation relative to tumor progression in high MHSS patients underscores its importance as a clinically meaningful event during systemic therapy.

### ML modeling

For ML analysis, we utilized KNIME version 4.5 (KNIME AG, Zurich, Switzerland), an open-source data analytics platform recognized for enabling reproducible ML workflows. The derivation dataset (n = 1262) consisted of patients treated in an earlier therapeutic era, primarily with sorafenib, and was used for model training and internal testing. The independent validation cohort (n = 764) included patients managed in subsequent eras when newer systemic therapies such as lenvatinib, nivolumab, and Atezo-bev became available. This approach allowed us to develop the MHSS in an earlier dataset and validate its generalizability in more contemporary treatment settings.

The ML modeling approach followed the methodology outlined in previous studies^22,23^. We evaluated several classification algorithms to predict the presence of CSPH, including logistic regression and tree-based ensemble models such as decision tree (DT), random forest (RF), gradient boosting machine (GBM), and eXtreme Gradient Boosting (XGBoost). All hyperparameters were kept at their default settings within the KNIME environment. Default hyperparameters were used for the RF (number of trees = 100, maximum depth = unlimited, split criterion = Gini impurity, bootstrap sampling enabled). Hyperparameter tuning via grid search was initially attempted; however, performance gains were negligible (<0.01 AUROC improvement) while overfitting risk increased. Model performance was assessed by comparing metrics such as accuracy, precision, recall, F1-score, and the area under the receiver operating characteristic curve (AUROC). For model interpretability, SHAP values were computed, and corresponding summary plots were generated to visualize the contribution of individual features to the prediction outcomes. Predicted probabilities by RF model for CSPH were calculated in the validation cohort, and patients were further stratified into risk groups using a probability threshold of 0.72, which maximized both the hazard ratio (HR) and p value between the groups using Youden’s index and receiver operating characteristic curve analysis. To facilitate clinical application and external validation, we developed a web-based MHSS calculator freely accessible at https://tmznjf.github.io/MHSS/.

### Statistical analyses

Categorical variables were expressed as counts and percentages and compared using the chi-square test. Continuous variables were reported as medians with interquartile ranges (IQR) and compared using the Mann–Whitney U test. Kaplan-Meier survival curves were compared across subgroups using the log-rank test to evaluate differences in OS and incident HD. Cox proportional hazards models were applied to identify HR for HD, VB, and OS. The AUROC values for different predictive models were compared using DeLong’s test. For PSM, nearest-neighbor matching was performed using a caliper width of 0.2 standard deviations of the propensity score. Balance was assessed using standardized mean differences (SMD), with SMD < 0.1 considered well-balanced. Kaplan-Meier curves were generated for HD, VB, and OS, and compared using Cox proportional hazards regression. All statistical analyses were performed using R version 4.0.3 (R Foundation for Statistical Computing, Vienna, Austria) and GraphPad Prism version 8 (GraphPad Software, San Diego, CA, USA).

## Supplementary information

Supplementary Information

## Data Availability

The minimal dataset that would be necessary to interpret, replicate, and build upon the methods or findings reported in the article is included in the article and its supplementary material. Further inquiries regarding data access can be directed at the corresponding author.
