# Supplementary material for: Machine learning based hepatic safety score predicts decompensation in hepatocellular carcinoma systemic therapy
Source: NPJ Digit Med. 2026 May 22;9:623. doi: 10.1038/s41746-026-02802-3 (PMC13470065; doi:10.1038/s41746-026-02802-3)
Supplement: Supplementary file 1 [file 41746_2026_2802_MOESM1_ESM.pdf]

Figure S1

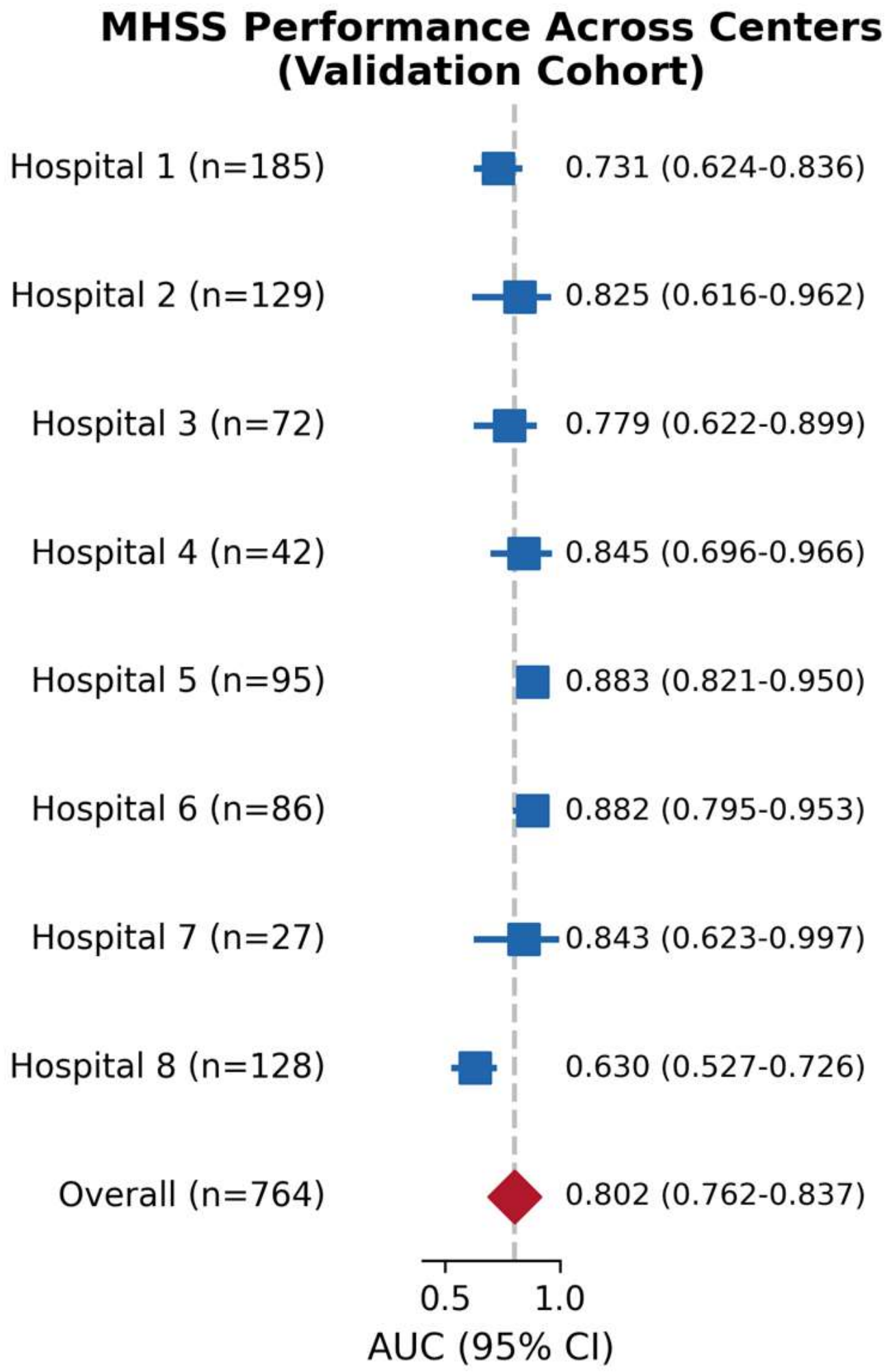

Figure S1. MHSS performance across centers. Forest plot showing AUC (95% CI) for CSPH prediction at each participating hospital in the validation cohort.

Figure S2

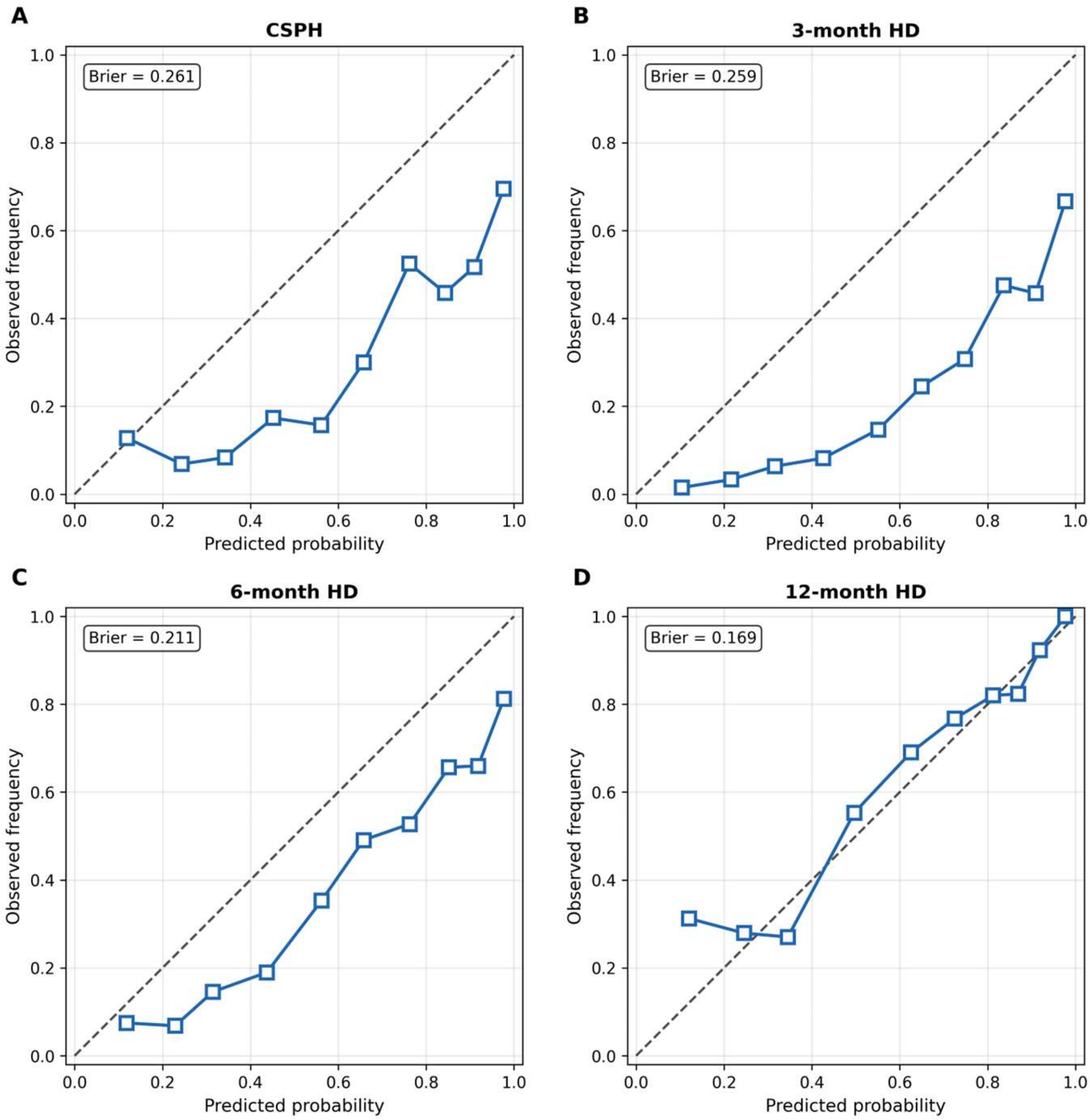

Figure S2. Calibration plots for MHSS in the validation cohort. Calibration curves showing the relationship between MHSS-predicted probability (x-axis) and observed frequency (y-axis) for (A) CSPH, (B) 3-month hepatic decompensation, (C) 6-month hepatic decompensation, and (D) 12-month hepatic decompensation. The dashed diagonal line represents perfect calibration.

Figure S3

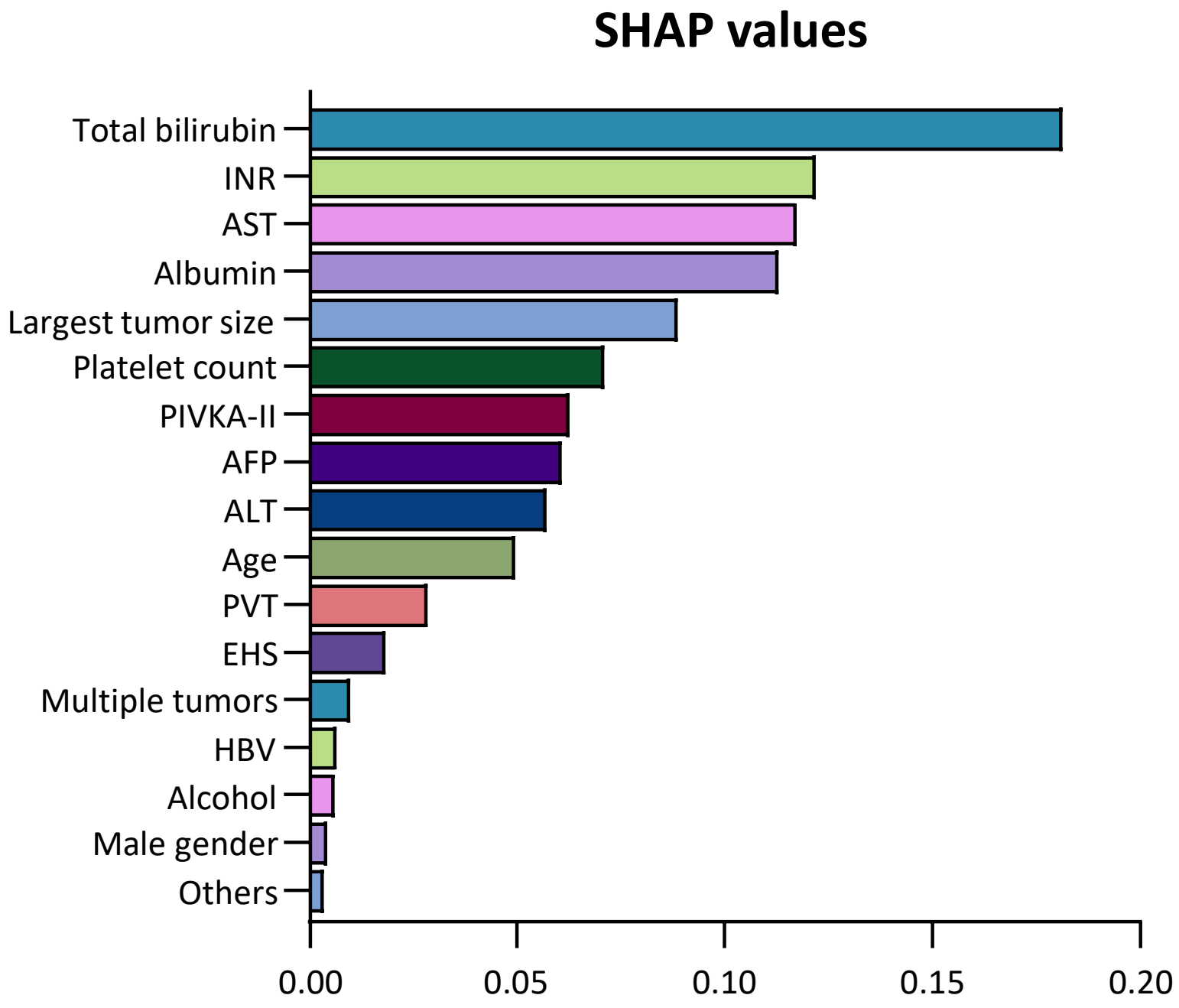

Figure S3. Feature importance plot for the random forest-based MHSS prediction model. SHAP values demonstrating the relative contribution of each clinical variable. PVT: Portal vein thrombosis; EHS: Extrahepatic spread; AST: Aspartate aminotransferase; ALT: Alanine aminotransferase; INR: International normalized ratio; AFP: Alpha-fetoprotein; PIVKA-II: Protein induced by vitamin K absence or antagonist-II; HBV: Hepatitis B virus; SHAP: SHapley Additive exPlanations; MHSS: Machine Learning-based Hepatic Safety Score.

Figure S4

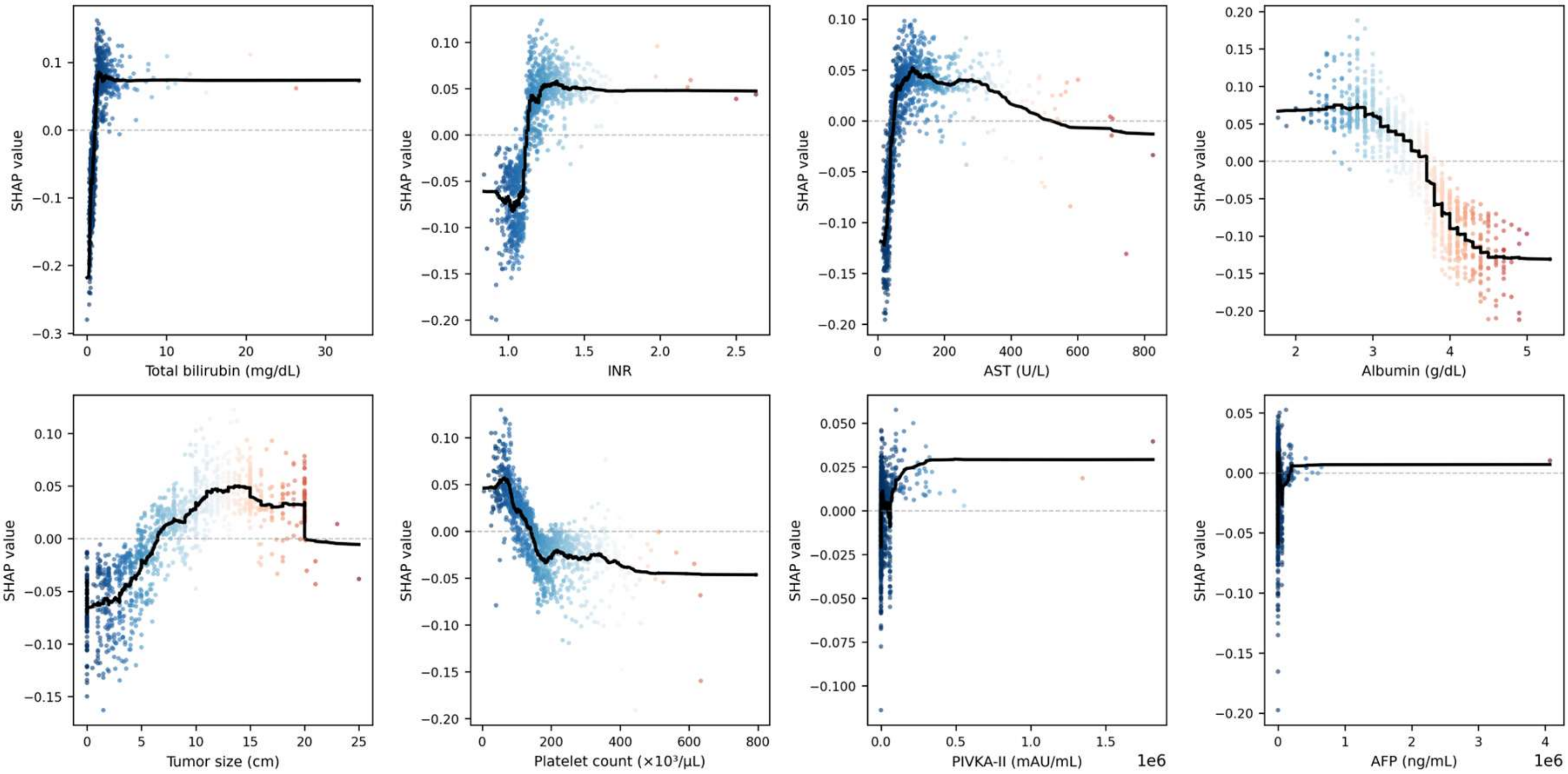

Figure S4. SHAP dependence plots for the top 8 features. Each plot shows the relationship between individual feature values (x-axis) and their SHAP values (y-axis), indicating how each feature contributes to MHSS predictions. Positive SHAP values increase the predicted CSPH risk, while negative values decrease it. The trend line and correlation coefficient (r) indicate the direction and strength of each feature's effect.

Figure S5

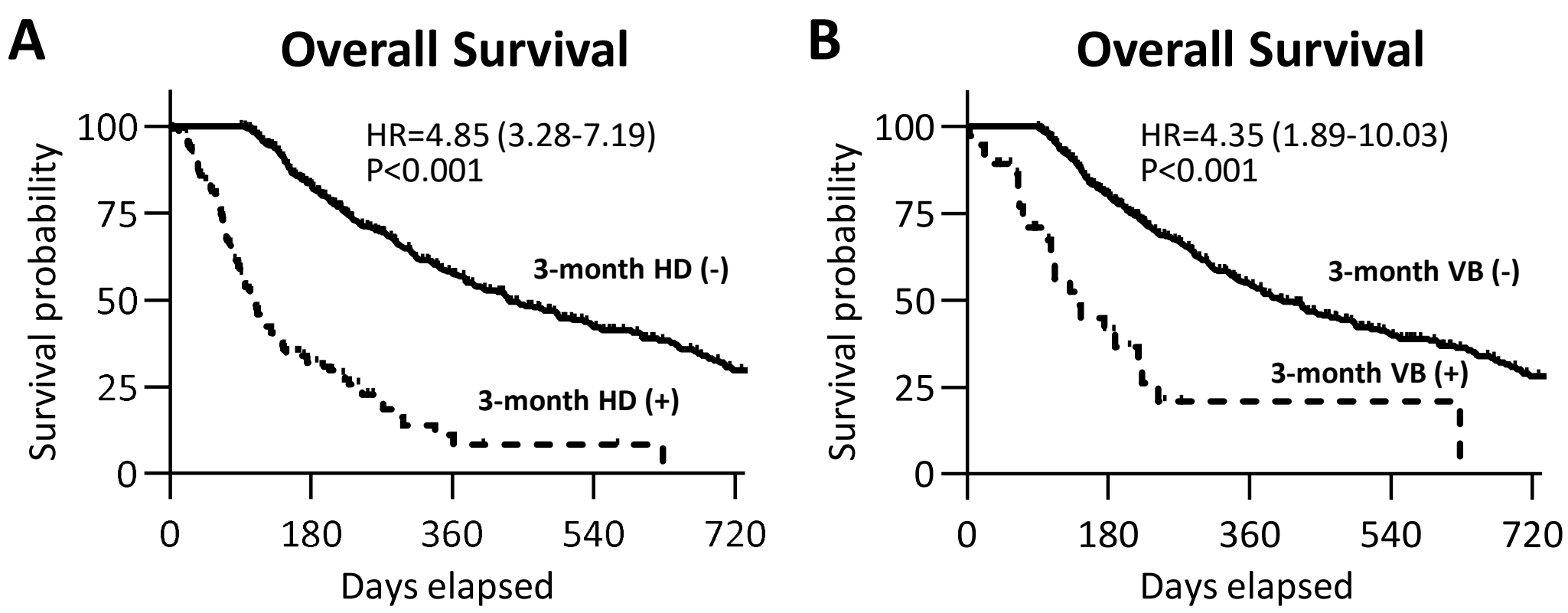

Figure S5. Association between early hepatic events and long-term survival. (A) Kaplan-Meier curves for overall survival comparing patients with versus without hepatic decompensation (HD) within 3 months. (B) Overall survival comparing patients with versus without variceal bleeding (VB) within 3 months. HD: Hepatic decompensation; VB: Variceal bleeding.

Figure S6

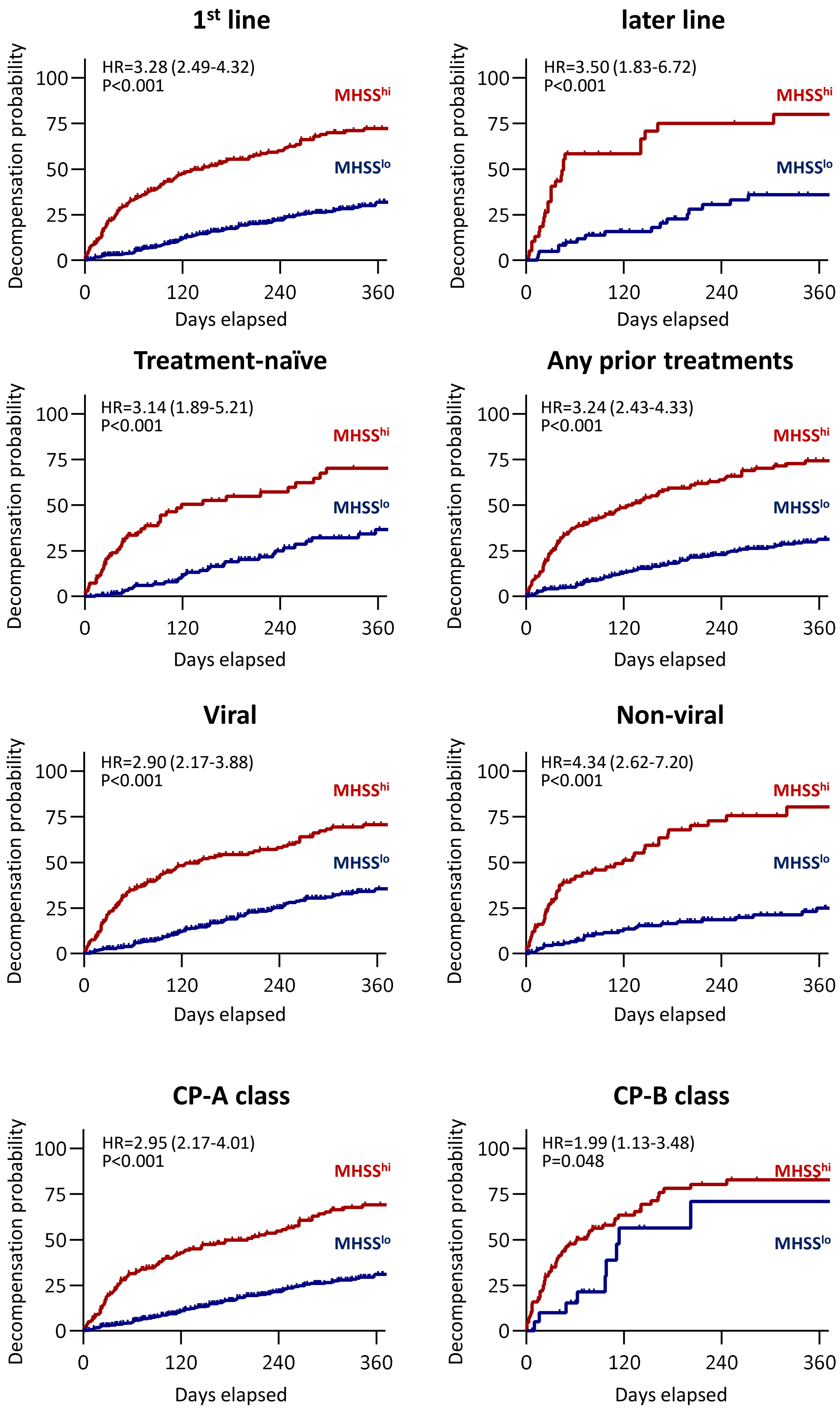

Figure S6. Subgroup analyses. Kaplan-Meier curves comparing incident decompensations between high MHSS ($MHSS^{hi}$) and low MHSS ($MHSS^{lo}$) groups among each subgroup.

Figure S7

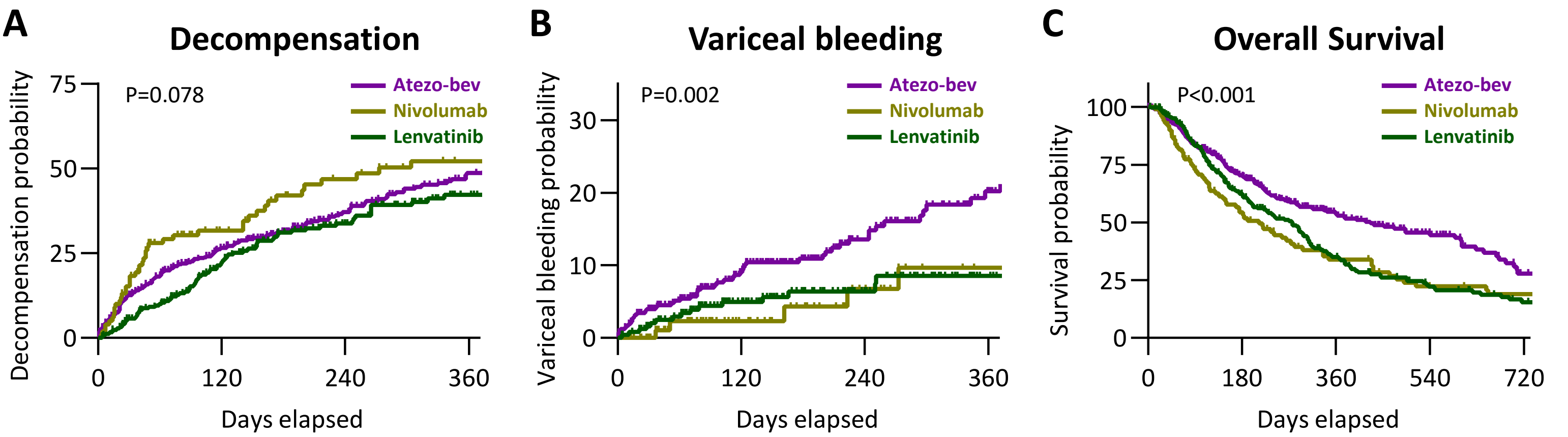

Figure S7. Comparison of clinical outcomes among systemic therapy regimens. Kaplan-Meier curves for (A) hepatic decompensation probability, (B) variceal bleeding probability, and (C) overall survival among patients treated with atezolizumab-bevacizumab (Atezo-bev), nivolumab, or lenvatinib as first-line therapy. Atezo-bev: Atezolizumab-bevacizumab.

Figure S8

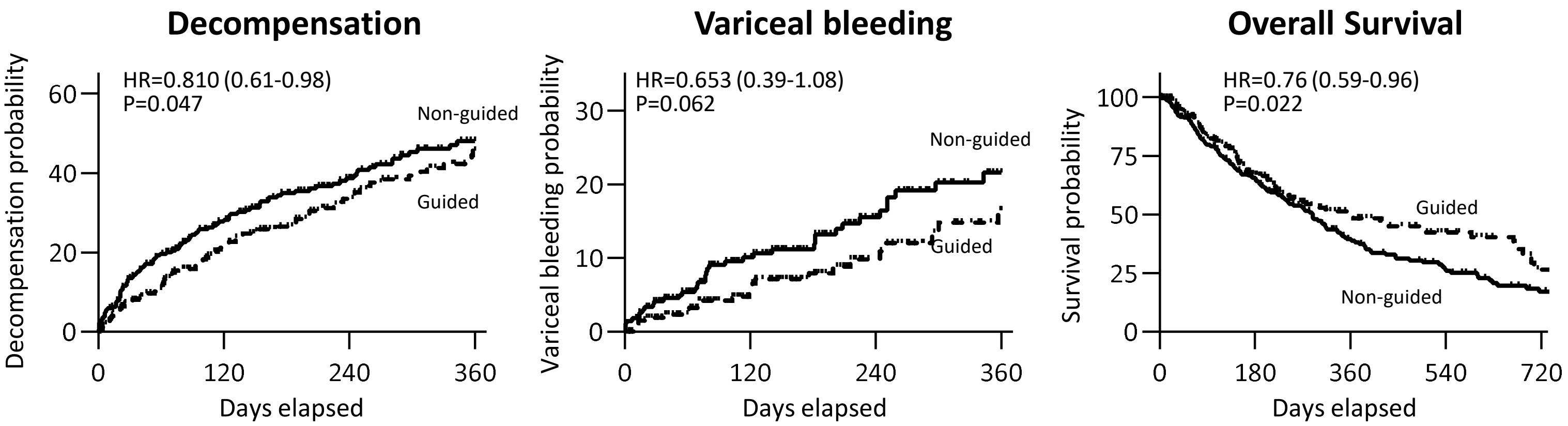

Figure S8. Simulation of the MHSS-guided treatment strategy in propensity-score matched cohort. Kaplan-Meier curves comparing clinical outcomes between MHSS-guided and non-guided treatment approaches for hepatic decompensation, variceal bleeding, and overall survival.

Figure S9

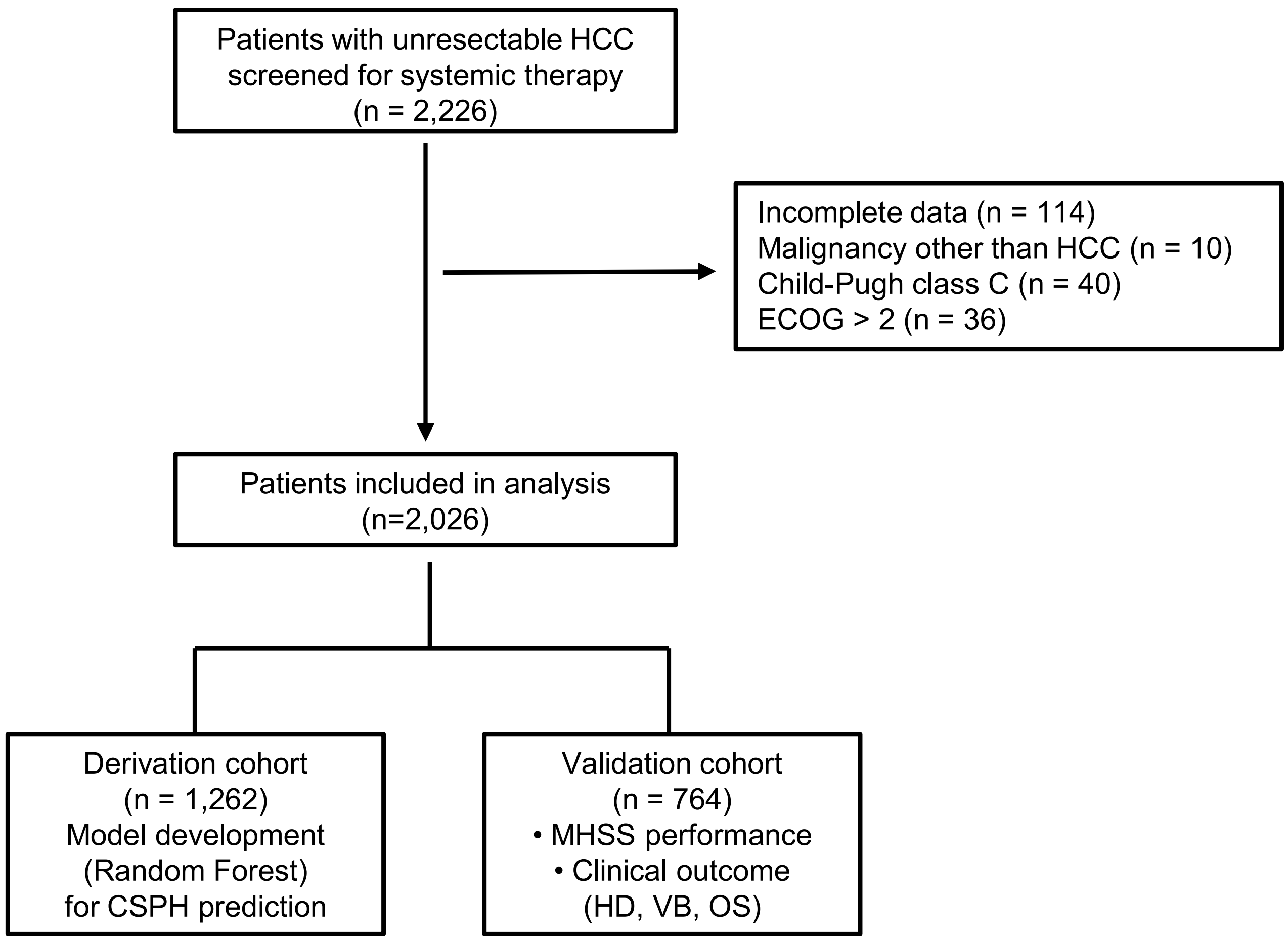

Figure S9. Study flowchart. HCC, hepatocellular carcinoma; ECOG, Eastern Cooperative Oncology Group; CSPH, clinically significant portal hypertension; MHSS, machine learning-based hepatic safety score; HD, hepatic decompensation; VB, variceal bleeding; OS, overall survival; Atezo-bev, atezolizumab-bevacizumab.

Table S1. Baseline Characteristics.

	Cohort 1 (n=1,262)	Cohort 2 (n=764)
Male sex	1,059 (83.9%)	648 (84.8%)
Age, years	61 (54-69)	64 (56-72)
Etiology		
HBV	874 (69.3%)	494 (64.7%)
HCV	91 (7.2%)	55 (7.2%)
Alcohol	332 (26.3%)	209 (27.4%)
Others	96 (7.6%)	103 (13.5%)
Largest intrahepatic size, cm	8.0 (4.0-12.5)	6.0 (2.6-11.0)
Multiple tumors	875 (69.3%)	597 (78.1%)
PVT	827 (65.5%)	343 (45.1%)
EHS	812 (64.3%)	490 (64.2%)
AST, U/L	70 (41-121)	50.5 (32-96)
ALT, U/L	34 (21-56)	28 (19-47)
Total bilirubin, mg/dL	1.0 (0.7-1.7)	0.8 (0.5-1.3)
Albumin, g/dL	3.5 (3.1-3.9)	3.7 (3.4-4.0)
Platelet, $\times 10^3/\mu L$	150 (103-218)	155 (107-219)
INR	1.15 (1.07-1.24)	1.10 (1.05-1.19)
AFP, ng/mL	614.1 (27.1-8,206.0)	351.2 (11.5-7,493.0)
PIVKA-II, mAU/mL	2,536.2 (204.0-17,764.5)	2,071.3 (123.0-16,418.0)
CSPH	911 (72.2%)	465 (60.9%)
Treatment-naïve	338 (26.7%)	202 (26.4%)

Data are presented as n (%) for categorical variables and median (interquartile range) for continuous variables. PVT: Portal vein thrombosis; EHS: Extrahepatic spread; AST: Aspartate aminotransferase; ALT: Alanine aminotransferase; INR: International normalized ratio; AFP: Alpha-fetoprotein; PIVKA-II: Protein induced by vitamin K absence or antagonist-II; CSPH: Clinically significant portal hypertension.

Table S2. Diagnostic Accuracies of Machine-Learning Algorithms for Clinically Significant Portal Hypertension.

	Recall	Precision	Sensitivity	Specificity	F1 score	Accuracy	AUROC	AUROC (validation)
Logistic regression	0.866	0.764	0.866	0.295	0.812	0.709	0.581	0.589
Decision tree	0.817	0.821	0.817	0.532	0.819	0.739	0.678	0.668
Random forest	0.902	0.827	0.902	0.504	0.863	0.792	**0.840**	**0.802**
Gradient boosting	0.910	0.824	0.910	0.489	0.865	0.794	0.836	0.781
XGBoost	0.907	0.834	0.907	0.525	0.869	0.802	0.839	0.771

AUROC: Area under the receiver operating characteristic curve.

Table S3. Propensity Score Matching Analysis for Tumor Burden Between MHSS Groups.

Variable	Low MHSS (n=233)	High MHSS (n=233)	SMD	p-value
Age, years	63.5 ± 11.8	62.1 ± 10.7	0.120	0.215
Male sex, n (%)	196 (84.1)	203 (87.1)	0.087	0.428
Tumor size, cm	7.5 (3.5-11.5)	7.8 (4.0-12.0)	0.046	0.623
Portal vein thrombosis, n (%)	126 (54.1)	136 (58.4)	0.086	0.401
Extrahepatic spread, n (%)	139 (59.7)	134 (57.5)	0.043	0.707
Multiple tumors, n (%)	199 (85.4)	201 (86.3)	0.025	0.894
AFP	811 (32-20218)	665 (53-8386)	0.027	0.768
**Outcome following PSM**	**HR (95% CI)**			**p-value**
Hepatic Decompensation	1.77 (1.34-2.34)			<0.001
Variceal Bleeding	1.91 (1.10-3.30)			0.021
Overall Survival	1.11 (0.86-1.42)			0.417

MHSS, machine learning-based hepatic safety score; PSM, propensity score matching; SMD, standardized mean difference; HR, hazard ratio; CI, confidence interval; AFP, alpha-fetoprotein.

Table S4. Baseline Characteristics by MHSS Status and Treatment Group.

	Low MHSS				High MHSS			
	Lenvatinib (n=151)	Nivolumab (n=62)	Atezo-bev (n=262)	p-value	Lenvatinib (n=101)	Nivolumab (n=38)	Atezo-bev (n=150)	p-value
Male sex	124/151 (82.1%)	48/62 (77.4%)	227/262 (86.6%)	0.104	88/101 (87.1%)	32/38 (84.2%)	129/150 (86.0%)	0.843
Age, years	64.0 (57.0-73.0)	58.5 (54.0-67.0)	66.0 (59.0-74.0)	0.053	61.0 (54.0-69.0)	58.0 (52.0-66.0)	63.0 (56.0-71.0)	0.156
Etiology								
HBV	97/151 (64.2%)	45/62 (72.6%)	161/262 (61.5%)	0.137	68/101 (67.3%)	26/38 (68.4%)	97/150 (64.7%)	0.831
HCV	15/151 (9.9%)	3/62 (4.8%)	13/262 (5.0%)	0.089	8/101 (7.9%)	4/38 (10.5%)	12/150 (8.0%)	0.863
Alcohol	33/151 (21.9%)	17/62 (27.4%)	64/262 (24.4%)	0.612	35/101 (34.7%)	15/38 (39.5%)	45/150 (30.0%)	0.463
Others	19/151 (12.6%)	7/62 (11.3%)	51/262 (19.5%)	0.074	3/101 (3.0%)	2/38 (5.3%)	21/150 (14.0%)	0.004
Largest intrahepatic size, cm	3.7 (1.2-9.0)	3.6 (1.8-6.8)	6.0 (2.5-10.0)	0.012	9.5 (4.5-13.0)	7.5 (3.8-12.0)	9.0 (4.0-13.0)	0.512
Multiple tumors	103/151 (68.2%)	49/62 (79.0%)	189/262 (72.1%)	0.288	91/101 (90.1%)	33/38 (86.8%)	132/150 (88.0%)	0.787
PVT	43/151 (28.5%)	10/62 (16.1%)	98/262 (37.4%)	0.005	68/101 (67.3%)	22/38 (57.9%)	102/150 (68.0%)	0.524
EHS	111/151 (73.5%)	52/62 (83.9%)	175/262 (66.8%)	0.033	48/101 (47.5%)	23/38 (60.5%)	81/150 (54.0%)	0.254
AST, U/L	42.0 (28.0-71.0)	37.5 (30.0-49.0)	40.0 (28.0-68.0)	0.544	78.0 (51.0-124.0)	82.5 (56.0-131.0)	81.0 (52.0-128.0)	0.892
ALT, U/L	27.0 (17.0-42.0)	27.0 (18.0-41.0)	24.0 (16.0-41.0)	0.721	35.0 (22.0-56.0)	38.0 (25.0-59.0)	36.0 (23.0-58.0)	0.743
Total bilirubin, mg/dL	0.7 (0.5-1.0)	0.5 (0.4-0.8)	0.6 (0.5-0.8)	0.021	1.4 (1.0-1.9)	1.2 (0.9-1.6)	1.4 (1.0-1.8)	0.234
Albumin, g/dL	3.9 (3.6-4.3)	3.8 (3.4-4.1)	3.9 (3.5-4.2)	0.234	3.5 (3.2-3.7)	3.6 (3.3-3.8)	3.5 (3.2-3.7)	0.445
Platelet, $\times 10^3/\mu L$	168.0 (122.0-234.0)	155.0 (119.0-188.0)	178.5 (127.0-242.0)	0.332	125.0 (84.0-201.0)	138.0 (92.0-215.0)	127.0 (85.0-205.0)	0.676
INR	1.1 (1.0-1.1)	1.1 (1.0-1.1)	1.1 (1.0-1.1)	0.567	1.2 (1.1-1.3)	1.1 (1.1-1.2)	1.2 (1.1-1.3)	0.123
AFP, ng/mL	61.3 (6.4-2443.8)	103.9 (6.0-2050.1)	133.5 (6.8-3061.0)	0.423	1205.0 (89.0-12450.0)	2156.0 (145.0-15680.0)	1689.0 (123.0-14230.0)	0.567
PIVKA-II, mAU/mL	493.7 (48.1-7459.0)	2382.4 (297.0-15920.0)	816.4 (69.0-8322.6)	0.188	5124.0 (512.0-29870.0)	6890.0 (723.0-34560.0)	5823.0 (634.0-31240.0)	0.689

Data are presented as n (%) for categorical variables and median (interquartile range) for continuous variables. PVT: Portal vein thrombosis; EHS: Extrahepatic spread; AST: Aspartate aminotransferase; ALT: Alanine aminotransferase; INR: International normalized ratio; AFP: Alpha-fetoprotein; PIVKA-II: Protein induced by vitamin K absence or antagonist-II.

Table S5. Multivariate Cox Regression for Overall Survival in Low MHSS Patients (Reference: Atezo-bev)

**Model**	**Variable**	**HR (95% CI)**	**p-value**
Univariate	Lenvatinib vs Atezo-bev	1.65 (1.23-2.21)	<0.001
Univariate	Nivolumab vs Atezo-bev	1.89 (1.32-2.71)	<0.001
Multivariate (Tumor burden + treatment duration)	Lenvatinib vs Atezo-bev	1.91 (1.37-2.66)	<0.001
Multivariate (Tumor burden + treatment duration)	Nivolumab vs Atezo-bev	2.18 (1.46-3.24)	<0.001

Adjusted for tumor size, portal vein thrombosis, extrahepatic spread, multiple tumors, and AFP, and treatment duration.

Table S6. Baseline Characteristics by ML-guided and non-guided groups, and Propensity-score matching.

Variable	Before: ML-guided (n=363)	Before: Non-guided (n=300)	Before: SMD	Before: p-value	After: ML-guided (n=271)	After: Non-guided (n=271)	After: SMD	After: p-value
Age, years	64.7 ± 11.6	63.4 ± 11.5	0.117	64.4 ± 11.3	63.6 ± 11.9	0.063	0.062	0.341
Male sex, n (%)	318 (87.6)	254 (84.7)	0.085	230 (84.9)	232 (85.6)	0.021	0.327	0.904
Portal vein thrombosis, n (%)	160 (44.1)	156 (52.0)	0.159	130 (48.0)	135 (49.8)	0.037	0.051	0.731
Extrahepatic spread, n (%)	224 (61.7)	182 (60.7)	0.021	171 (63.1)	164 (60.5)	0.053	0.846	0.596
Largest tumor size, cm	6.5 (3.0-11.2)	6.5 (2.6-11.5)	<0.001	6.0 (2.7-10.1)	6.0 (2.7-11.4)	0.077	0.846	0.522
Multiple tumors, n (%)	279 (76.9)	231 (77.0)	0.003	206 (76.0)	209 (77.1)	0.026	1.000	0.839
AST, U/L	47.0 (30.0-85.5)	62.5 (35.0-105.2)	0.123	51.0 (30.0-101.5)	59.0 (34.5-102.5)	0.008	0.003	0.177
ALT, U/L	27.0 (18.0-46.5)	30.0 (19.8-49.2)	0.053	28.0 (19.0-49.0)	30.0 (19.0-50.0)	0.015	0.067	0.653
Total bilirubin, mg/dL	0.72 (0.50-1.17)	0.94 (0.63-1.39)	0.196	0.72 (0.52-1.12)	0.90 (0.61-1.34)	0.113	<0.001	0.001
Albumin, g/dL	3.7 ± 0.5	3.7 ± 0.5	0.085	3.7 ± 0.5	3.7 ± 0.5	0.040	0.131	0.416
Platelet, ×10³/μL	161.0 (111.0-224.5)	160.0 (105.2-229.2)	0.019	160.0 (112.0-237.5)	166.0 (106.5-234.0)	0.011	0.524	0.625
INR	1.10 (1.04-1.18)	1.10 (1.05-1.21)	0.126	1.10 (1.04-1.18)	1.10 (1.05-1.19)	0.036	0.079	0.377
AFP, ng/mL	288 (8-4492)	288 (18-11226)	0.036	260 (8-5828)	238 (16-8626)	0.069	0.305	0.707
PIVKA-II, mAU/mL	1191 (82-14866)	2278 (120-16817)	0.061	1409 (103-19077)	1910 (98-14020)	0.017	0.261	0.993
HBV, n (%)	228 (62.8)	193 (64.3)	0.032	180 (66.4)	180 (66.4)	<0.001	0.746	1.000
HCV, n (%)	22 (6.1)	27 (9.0)	0.112	21 (7.7)	17 (6.3)	0.058	0.197	0.614
Alcohol, n (%)	94 (25.9)	88 (29.3)	0.077	76 (28.0)	77 (28.4)	0.008	0.368	1.000
MASLD/Other, n (%)	60 (16.5)	31 (10.3)	0.182	27 (10.0)	31 (11.4)	0.048	0.028	0.677
High MHSS, n (%)	101 (27.8)	149 (49.7)	0.460	56 (20.7)	130 (48.0)	0.601	<0.001	<0.001
Hepatic decompensation, n (%)	136 (37.5)	131 (43.7)	0.127	100 (36.9)	115 (42.4)	0.113	0.123	0.219

SMD, standardized mean difference; AST, aspartate aminotransferase; ALT, alanine aminotransferase; AFP, alpha-fetoprotein; PIVKA-II, protein induced by vitamin K absence-II; HBV, hepatitis B virus; HCV, hepatitis C virus; MASLD, metabolic dysfunction-associated steatotic liver disease; MHSS, machine learning-based hepatic safety score. Continuous variables are presented as mean ± SD or median (interquartile range) according to data distribution. Categorical variables are presented as n (%).
